# (1-Hy­droxy­ethyl­idene)(meth­yl)aza­nium bromide–*N*-methyl­acetamide (1/1)

**DOI:** 10.1107/S1600536812016984

**Published:** 2012-04-21

**Authors:** Bin Wei

**Affiliations:** a Ordered Matter Science Research Center, Southeast University, Nanjing 211189, People’s Republic of China

## Abstract

The asymmetric unit of the organic hybrid salt, C_3_H_8_NO^+^·Br^−^·C_3_H_7_NO, comprises an *N*-methyl­acetamide cation, a *N*-methyl­acetamide mol­ecule and a bromide anion. The amide species are linked head-to-head through a short O⋯H⋯O hydrogen bond, giving a monocation, which is extended by N—H⋯Br hydrogen bonds into chains along the *b*-axis direction.

## Related literature

For general background to frameworks and structural phase transitions, see: Ye *et al.* (2009 ▶); Zhang *et al.* (2009 ▶). For the structure of the hemihydro­chloride of *N*-methyl­acetamide, see: Jaber *et al.* (1983 ▶). 
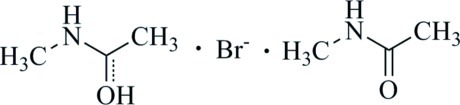


## Experimental

### 

#### Crystal data

C_3_H_8_NO^+^·Br^−^·C_3_H_7_NO
*M* *_r_* = 227.11
Orthorhombic, 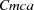

*a* = 6.8830 (14) Å
*b* = 23.029 (5) Å
*c* = 13.291 (3) Å
*V* = 2106.7 (8) Å^3^
*Z* = 8
Mo *K*α radiation
μ = 3.87 mm^−1^
*T* = 298 K
0.20 × 0.20 × 0.20 mm

#### Data collection

Rigaku SCXmini diffractometer
Absorption correction: multi-scan (*CrystalClear*; Rigaku, 2005 ▶) *T*_min_ = 0.461, *T*_max_ = 0.480
10344 measured reflections
1311 independent reflections
858 reflections with *I* > 2σ(*I*)
*R*_int_ = 0.073

#### Refinement

*R*[*F* ^2^ > 2σ(*F* ^2^)] = 0.044
*wR*(*F* ^2^) = 0.101
*S* = 1.06
1311 reflections
80 parameters
H atoms treated by a mixture of independent and constrained refinement
Δρ_max_ = 0.40 e Å^−3^
Δρ_min_ = −0.25 e Å^−3^

### 

Data collection: *CrystalClear* (Rigaku, 2005 ▶); cell refinement: *CrystalClear*; data reduction: *CrystalClear*; program(s) used to solve structure: *SHELXTL* (Sheldrick, 2008 ▶); program(s) used to refine structure: *SHELXTL*; molecular graphics: *SHELXTL*; software used to prepare material for publication: *SHELXTL*.

## Supplementary Material

Crystal structure: contains datablock(s) I, global. DOI: 10.1107/S1600536812016984/zs2188sup1.cif

Structure factors: contains datablock(s) I. DOI: 10.1107/S1600536812016984/zs2188Isup2.hkl

Supplementary material file. DOI: 10.1107/S1600536812016984/zs2188Isup3.cml

Additional supplementary materials:  crystallographic information; 3D view; checkCIF report

## Figures and Tables

**Table 1:** Hydrogen-bond geometry (Å, °)

*D*—H⋯*A*	*D*—H	H⋯*A*	*D*⋯*A*	*D*—H⋯*A*
N2—H2⋯Br1^i^	0.89 (5)	2.51 (5)	3.402 (5)	178 (5)
O1—H3⋯O2	1.16 (7)	1.27 (7)	2.437 (4)	179 (6)
N1—H1⋯Br1	0.83 (4)	2.48 (5)	3.304 (4)	174 (5)

Symmetry code: (i) 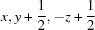.

**Fig. 1.**
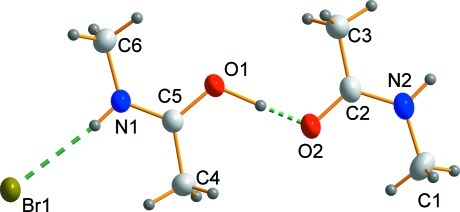
The molecular structure of the title compound, with the inter-species hydrogen bonds shown as dashed lines. Displacement ellipsoids are drawn at the 30% probability level.

**Fig. 2.**
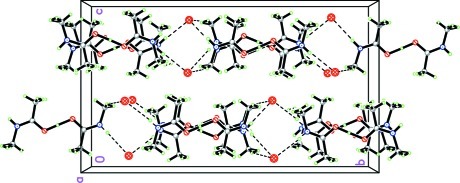
A view of the packing of the title compound, showing the hydrogen-bonded chain extension along the b axis. Dashed lines indicate hydrogen bonds.

## supplementary crystallographic information

### Comment

Recent studies have revealed that small molecular compounds which have one or more amidogens may possess dielectric-ferroelectric properties (Ye *et al.*, 2009; Zhang *et al.*, 2009). Our research has been aimed at the synthesis of aromatic amidogen-containing compounds which may possess these properties. As part of our ongoing studies, we report here the crystal structure of the title compound, C_6_H_15_N_2_^+^ Br^-^, the hydrobromide of *N*-methylacetamide The structure of the analogous hydrochloride of *N*-methylacetamide has previously been reported (Jaber *et al.*, 1983).

The structure of the title compound, determined at ambient temperature (298 K), reveals that the asymmetric unit contains an *N*-methylacetamide cation, a *N*-methylacetamide molecule and a bromide anion (Fig. 1). The transferred proton is found within a short O1···H···O2 hydrogen bond (Table 1) linking the two molecules head-to-head in the monocation. These cations and the bromide anions form N—H···Br hydrogen-bonding associations giving one-dimensional chains which extend along the *b*-cell direction (Fig. 2). Unfortunately, the dielectric constant of the title compound as a function of temperature indicates that the permittivity is basically temperature-independent below the melting point (368-369 K) and that it has no dielectric disuniform from 80 K to 405 K.

### Experimental

The *N*-methylacetamide(1.46 g, 20 mmol) and hydrobromic acid (1.62 g, 20 mmol) was combined in 30 ml of aqueous solution. The mixture was stirred for 30 min to allow complete reaction and good quality blocky single crystals were obtained by slow evaporation after *ca.* two weeks (yield, 42%)

### Refinement

The H atoms on the amide groups and the H within the short intramolecular O···H···O hydrogen bond were located in difference-Fourier analysis and their positional and isotropic displacement parameters were refined. The methyl H atoms were placed in geometrically idealized positions and constrained to ride on their parent atoms with C—H = 0.96 Å and with *U*_iso_(H) = 1.5*U*_eq_(C).

### Crystal data

**Table:**

C_3_H_8_NO^+^·Br^−^·C_3_H_7_NO	*F*(000) = 928
*M**_r_* = 227.11	*D*_x_ = 1.432 Mg m^−^^3^
Orthorhombic, *C**m**c**a*	Mo *K*α radiation, λ = 0.71073 Å
Hall symbol: -C 2bc 2	Cell parameters from 3638 reflections
*a* = 6.8830 (14) Å	θ = 3.0–27.5°
*b* = 23.029 (5) Å	µ = 3.87 mm^−^^1^
*c* = 13.291 (3) Å	*T* = 298 K
*V* = 2106.7 (8) Å^3^	Block, colorless
*Z* = 8	0.20 × 0.20 × 0.20 mm

### Data collection

**Table:**

Rigaku SCXmini diffractometer	858 reflections with *I* > 2σ(*I*)
Radiation source: fine-focus sealed tube	*R*_int_ = 0.073
Graphite monochromator	θ_max_ = 27.5°, θ_min_ = 3.1°
ω scans	*h* = −8→8
Absorption correction: multi-scan (*CrystalClear*; Rigaku, 2005)	*k* = −29→29
*T*_min_ = 0.461, *T*_max_ = 0.480	*l* = −17→17
10344 measured reflections	2 standard reflections every 150 reflections
1311 independent reflections	intensity decay: none

### Refinement

**Table:**

Refinement on *F*^2^	Primary atom site location: structure-invariant direct methods
Least-squares matrix: full	Secondary atom site location: difference Fourier map
*R*[*F*^2^ > 2σ(*F*^2^)] = 0.044	Hydrogen site location: inferred from neighbouring sites
*wR*(*F*^2^) = 0.101	H atoms treated by a mixture of independent and constrained refinement
*S* = 1.06	*w* = 1/[σ^2^(*F*_o_^2^) + (0.0445*P*)^2^ + 0.3335*P*] where *P* = (*F*_o_^2^ + 2*F*_c_^2^)/3
1311 reflections	(Δ/σ)_max_ < 0.001
80 parameters	Δρ_max_ = 0.40 e Å^−^^3^
0 restraints	Δρ_min_ = −0.25 e Å^−^^3^

### Special details

**Table:**

Geometry. All e.s.d.'s (except the e.s.d. in the dihedral angle between two l.s. planes) are estimated using the full covariance matrix. The cell e.s.d.'s are taken into account individually in the estimation of e.s.d.'s in distances, angles and torsion angles; correlations between e.s.d.'s in cell parameters are only used when they are defined by crystal symmetry. An approximate (isotropic) treatment of cell e.s.d.'s is used for estimating e.s.d.'s involving l.s. planes.
Refinement. Refinement of *F*^2^ against ALL reflections. The weighted *R*-factor *wR* and goodness of fit *S* are based on *F*^2^, conventional *R*-factors *R* are based on *F*, with *F* set to zero for negative *F*^2^. The threshold expression of *F*^2^ > σ(*F*^2^) is used only for calculating *R*-factors(gt) *etc*. and is not relevant to the choice of reflections for refinement. *R*-factors based on *F*^2^ are statistically about twice as large as those based on *F*, and *R*- factors based on ALL data will be even larger.

### Fractional atomic coordinates and isotropic or equivalent isotropic displacement parameters (Å^2^)

**Table:**

	*x*	*y*	*z*	*U*_iso_*/*U*_eq_	Occ. (<1)
Br1	0.5000	0.147343 (18)	0.08336 (4)	0.0651 (3)	
H1	0.5000	0.233 (2)	0.196 (4)	0.069 (16)*	
N1	0.5000	0.25929 (16)	0.2388 (3)	0.0553 (10)	
O1	0.5000	0.35425 (13)	0.2661 (3)	0.0714 (10)	
C2	0.5000	0.4921 (2)	0.2594 (4)	0.0578 (12)	
H2	0.5000	0.573 (2)	0.277 (4)	0.092 (19)*	
N2	0.5000	0.54618 (17)	0.2301 (3)	0.0635 (11)	
O2	0.5000	0.45173 (14)	0.1949 (3)	0.0717 (10)	
C5	0.5000	0.31197 (19)	0.2038 (3)	0.0506 (11)	
C6	0.5000	0.24321 (19)	0.3449 (3)	0.0658 (14)	
H7A	0.5414	0.2758	0.3846	0.099*	0.50
H7B	0.5874	0.2113	0.3552	0.099*	0.50
H7C	0.3712	0.2320	0.3647	0.099*	0.50
C4	0.5000	0.3231 (2)	0.0925 (3)	0.0695 (15)	
H8A	0.5940	0.3526	0.0769	0.104*	0.50
H8B	0.3734	0.3359	0.0719	0.104*	0.50
H8C	0.5326	0.2880	0.0575	0.104*	0.50
C3	0.5000	0.4790 (2)	0.3693 (4)	0.0724 (15)	
H9A	0.4649	0.5133	0.4061	0.109*	0.50
H9B	0.6273	0.4666	0.3894	0.109*	0.50
H9C	0.4078	0.4488	0.3831	0.109*	0.50
C1	0.5000	0.5647 (2)	0.1246 (4)	0.0804 (16)	
H10A	0.6291	0.5759	0.1053	0.121*	0.50
H10B	0.4138	0.5972	0.1165	0.121*	0.50
H10C	0.4571	0.5332	0.0828	0.121*	0.50
H3	0.5000	0.401 (3)	0.231 (5)	0.15 (3)*	

### Atomic displacement parameters (Å^2^)

**Table:**

	*U*^11^	*U*^22^	*U*^33^	*U*^12^	*U*^13^	*U*^23^
Br1	0.0934 (5)	0.0415 (3)	0.0606 (4)	0.000	0.000	−0.0013 (2)
N1	0.074 (3)	0.039 (2)	0.052 (2)	0.000	0.000	−0.001 (2)
O1	0.124 (3)	0.0397 (18)	0.051 (2)	0.000	0.000	−0.0049 (15)
C2	0.060 (3)	0.046 (3)	0.068 (3)	0.000	0.000	−0.005 (3)
N2	0.077 (3)	0.042 (2)	0.071 (3)	0.000	0.000	−0.007 (2)
O2	0.115 (3)	0.0394 (17)	0.061 (2)	0.000	0.000	−0.0052 (16)
C5	0.054 (3)	0.045 (3)	0.052 (3)	0.000	0.000	−0.002 (2)
C6	0.084 (4)	0.054 (3)	0.059 (3)	0.000	0.000	0.008 (2)
C4	0.099 (4)	0.057 (3)	0.052 (3)	0.000	0.000	0.003 (2)
C3	0.104 (4)	0.055 (3)	0.058 (3)	0.000	0.000	−0.003 (3)
C1	0.100 (4)	0.062 (3)	0.080 (4)	0.000	0.000	0.022 (3)

### Geometric parameters (Å, º)

**Table:**

N1—C5	1.299 (5)	C6—H7B	0.9600
N1—C6	1.458 (6)	C6—H7C	0.9600
N1—H1	0.83 (4)	C4—H8A	0.9600
O1—C5	1.278 (5)	C4—H8B	0.9600
O1—H3	1.16 (7)	C4—H8C	0.9600
C2—O2	1.266 (5)	C3—H9A	0.9600
C2—N2	1.304 (5)	C3—H9B	0.9600
C2—C3	1.491 (6)	C3—H9C	0.9600
N2—C1	1.466 (6)	C1—H10A	0.9600
N2—H2	0.89 (5)	C1—H10B	0.9600
C5—C4	1.502 (6)	C1—H10C	0.9600
C6—H7A	0.9600		
			
C5—N1—C6	125.7 (4)	H7B—C6—H7C	109.5
C5—N1—H1	116 (4)	C5—C4—H8A	109.5
C6—N1—H1	118 (4)	C5—C4—H8B	109.5
C5—O1—H3	116 (3)	H8A—C4—H8B	109.5
O2—C2—N2	119.9 (5)	C5—C4—H8C	109.5
O2—C2—C3	121.0 (4)	H8A—C4—H8C	109.5
N2—C2—C3	119.0 (4)	H8B—C4—H8C	109.5
C2—N2—C1	124.3 (5)	C2—C3—H9A	109.5
C2—N2—H2	118 (4)	C2—C3—H9B	109.5
C1—N2—H2	118 (4)	H9A—C3—H9B	109.5
C2—O2—H3	115 (3)	C2—C3—H9C	109.5
O1—C5—N1	118.6 (4)	H9A—C3—H9C	109.5
O1—C5—C4	120.6 (4)	H9B—C3—H9C	109.5
N1—C5—C4	120.8 (4)	N2—C1—H10A	109.5
N1—C6—H7A	109.5	N2—C1—H10B	109.5
N1—C6—H7B	109.5	H10A—C1—H10B	109.5
H7A—C6—H7B	109.5	N2—C1—H10C	109.5
N1—C6—H7C	109.5	H10A—C1—H10C	109.5
H7A—C6—H7C	109.5	H10B—C1—H10C	109.5
			
C6—N1—C5—O1	0.00	C1—N2—C2—O2	0.00
C6—N1—C5—C4	180.00	C1—N2—C2—C3	180.00

### Hydrogen-bond geometry (Å, º)

**Table:**

*D*—H···*A*	*D*—H	H···*A*	*D*···*A*	*D*—H···*A*
N2—H2···Br1^i^	0.89 (5)	2.51 (5)	3.402 (5)	178 (5)
O1—H3···O2	1.16 (7)	1.27 (7)	2.437 (4)	179 (6)
N1—H1···Br1	0.83 (4)	2.48 (5)	3.304 (4)	174 (5)

Symmetry code: (i) *x*, *y*+1/2, −*z*+1/2.

## References

[bb1] Jaber, M., Guilhem, J. & Loiseleur, H. (1983). *Acta Cryst.* C**39**, 485–487.

[bb2] Rigaku (2005). *CrystalClear.* Rigaku Corporation, Tokyo, Japan.

[bb3] Sheldrick, G. M. (2008). *Acta Cryst.* A**64**, 112–122. 10.1107/S0108767307043930 18156677

[bb4] Ye, H.-Y., Fu, D.-W., Zhang, Y., Zhang, W., Xiong, R.-G. & Huang, S.-D. (2009). *J. Am. Chem. Soc.* **131**, 42–43. 10.1021/ja808331g 19128170

[bb5] Zhang, W., Cheng, L.-Z., Xiong, R.-G., Nakamura, T. & Huang, S.-D. (2009). *J. Am. Chem. Soc.* **131**, 12544–12545. 10.1021/ja905399x 19685869

